# Accuracy of Visual Scoring and Semi-Quantification of Ultrasound Strain Elastography – A Phantom Study

**DOI:** 10.1371/journal.pone.0088699

**Published:** 2014-02-12

**Authors:** Jonathan Frederik Carlsen, Caroline Ewertsen, Adrian Săftoiu, Lars Lönn, Michael Bachmann Nielsen

**Affiliations:** 1 Department of Radiology, Rigshospitalet Copenhagen University Hospital, Copenhagen, Denmark; 2 Research Center of Gastroenterology and Hepatology, University of Medicine and Pharmacy, Craiova, Romania; 3 Department of Endoscopy, Gastrointestinal Unit, Copenhagen University Hospital Herlev, Herlev, Denmark; 4 Department of Vascular Surgery, Rigshospitalet Copenhagen University Hospital, Copenhagen, Denmark; Semmelweis University, Hungary

## Abstract

**Purpose:**

The aim of this study was to evaluate the performance of strain elastography in an elasticity phantom and to assess which factors influenced visual scoring, strain histograms and strain ratios. Furthermore this study aimed to evaluate the effect of observer experience on visual scorings.

**Materials and Methods:**

Two operators examined 20 targets of various stiffness and size (16.7 to 2.5 mm) in an elasticity phantom at a depth of 3.5 cm with a 5–18 MHz transducer. Two pre-settings were used yielding 80 scans. Eight evaluators, four experienced, four inexperienced, performed visual scorings. Cut-offs for semi-quantitative methods were established for prediction of target stiffness. Data was pooled in two categories allowing calculations of sensitivity and specificity. *Statistical tests* chi-square test and linear regression as relevant.

**Results:**

Strain ratios and strain histograms were superior to visual scorings of both experienced and inexperienced observers (p = 0.025, strain histograms vs. experienced observers, p<0.001, strain histograms vs. inexperienced observers, p = 0.044 strain ratios vs. experienced observers and p = 0.002 strain ratios vs. inexperienced observers). No significant difference in predicting target stiffness between strain ratios and strain histograms (p = 0.83) nor between experienced and inexperienced observers (p = 0.054) was shown when using four categories. When pooling data in two groups (80 kPa/45 kPa vs. 14/8 kPa) the difference between the observers became significant (p<0.001). Target size had a significant influence on strain ratios measurements (p = 0.017) and on visual scorings (p<0.001) but not on the strain histograms(p = 0.358). Observer experience had significant effect on visual scorings(p = 0.003).

**Conclusion:**

Strain ratios and strain histograms are superior to visual scoring in assessing target stiffness in a phantom. Target size had a significant impact on strain ratios and visual scoring, but not on strain histograms. Experience influenced visual scorings but the difference between experienced and inexperienced observers was only significant when looking at two classes of target stiffness.

## Introduction

The principle of strain imaging was first reported in 1991 by Ophir and coworkers [1]. Strain elastography (SE) is one of several ultrasonography (US) based imaging modalities that estimate tissue stiffness [1]–[3]. SE has been suggested as a tool for predicting malignancy in focal lesions. Malignant lesions are in general stiffer than benign lesions, a feature well known from manual palpation of superficial tumors [4]. SE-measurements are not directly quantifiable, thus several qualitative and semi-quantitative methods have been proposed and investigated in clinical trials.

Strain is inversely proportional to lesion stiffness. In SE the calculated strain is color coded by the software and displayed as a transparent overlay on the gray scale ultrasonography images. Itoh et al. proposed a five point scoring system for evaluation of malignancy in breast tumors by assessing lesion color [5]. This qualitative scoring system and other similar visual scoring systems have been applied in studies on breast cancer diagnosis [6]–[8] as well as in lymph-node diagnosis [9], [10], thyroid nodule diagnosis [11], [12], and in the diagnosis of non-nodal neck masses [13]. Interobserver agreement of visual scoring has been investigated in different clinical fields and in a single phantom study [9], [11], [14], [15]. The interobserver agreements reported in these studies varied from average to very good. A method of semi-quantification applied in SE is the calculation of strain-ratios [3], [16]. Strain-ratios are calculated using two regions of interest (ROIs), one in the lesion and one in the surrounding tissue. Hard lesions have high strain-ratios (>1) and soft lesions have low strain-ratios (<1). Strain-ratios have been used in different applications such as, breast cancer diagnosis [17], [18], pancreatic mass evaluation [19], classification of liver fibrosis [20], and for the prediction of optimal biopsy targets in prostate cancer diagnosis [21]. Another method of SE semi-quantification, recently proposed by Săftoiu et al. [22], is hue-histogram analysis [3]. The hue-histogram or strain-histogram is an orderly depiction of the quantity of the 256 colors in the elastogram. Mean pixel color value corresponds to the overall stiffness of the lesion. Strain-histogram analysis has previously been evaluated in the diagnosis of pancreatic masses [23], steato-fibrosis [24], breast cancer [25], and enlarged gastrointestinal lymph nodes [22], [26].

The primary aim of this study was to evaluate the ability of visual scoring, strain-ratios and strain-histograms to predict the stiffness of cylinders of known stiffness in a commercially available elasticity tissue-mimicking phantom. Furthermore, the aim was to assess which factors influenced the results of the methods of SE-evaluation including the impact of evaluator experience for the visual scorings.

## Materials and Methods

### Phantom

**Figure 1:**
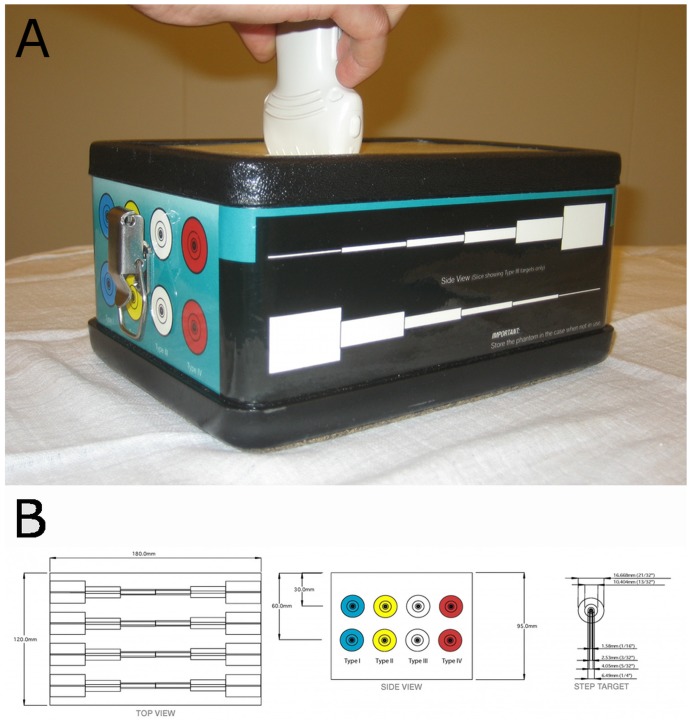
The Elasticity Phantom. 1a. The study setup with the phantom and US transducer. (Elasticity QA, model 049A, CIRS (CIRS, Virginia, USA)). 1b. Schematic representation of the elasticity phantom used. (Elasticity QA, model 049A, CIRS (CIRS, Virginia, USA))

We used an elasticity phantom (Elasticity QA, model 049A, CIRS (CIRS, Virginia, USA))(Figure 1a), which consisted of eight cylinders enclosed in a surrounding medium (Figure 1b). Both cylinders and surrounding medium were made from Zerdine®, a transparent polyacrylamide polymer (US. pat. 5.196.343, 1993). The cylinders were placed in two layers. We used only the superficial layer, with the center of the cylinders at 3.5 cms depth. Each cylinder had one of four different levels stiffness ranging from soft to hard (8, 14, 45 and 80 kPa). The background material had a stiffness of 25 kPa. The speed of sound was 1540 m/s and the attenuation was 0.5 dB/cm-MHz, the phantom characteristics thus being comparable to breast tissue [27]. The diameter of each cylinder decreased stepwise along the axis of the cylinder. We used the five largest diameters (16.7, 10.4, 6.5, 4.1 and 2.5 mm) of the phantom, as smaller diameters were impossible to discern in either B-mode or SE. This added up to 20 targets evaluated in this study.

### Ultrasonography

Two physicians (JFC, MBN) independently recorded all elastography examinations independently with a Hitachi Ascendus system (Hitachi, Tokyo, Japan) using an L75-probe with a bandwidth of 5-18 MHz. Scans were performed perpendicular to the phantom cylinder axes, and the transducer was coupled to the surface by ultrasound gel. Video clips of five seconds were stored for later analysis. All images were labeled with pre-assigned letter codes to facilitate the blinded operator evaluation. Two different elastography pre-settings, 1 and 2, which varied in their color distribution on the elastogram, were used. The differences in elastography parameters between the two settings can be seen in table 1. A manual compression rate of 100/minute was achieved by using a digital metronome. The compression quality was monitored by a strain-monitor on the scanner. Lesions covered a range of 25% to 50% of the elastography box except in the two smallest targets where this was impossible, thus the box was sized as small as possible. Absolute measures of the boxes were not recorded. The two physicians scanned all targets with one pre-setting at a time.

**Table 1:** An Overview of the Differences in Elasticity Parameters Between Pre-setting 1 and 2.

	Pameter range	Presetting 1*	Presetting 2#	Explanation of parameter
Elasticity Dynamic Range (eDR)	1–8	1	4	Changes the dynamic range of elastography images. Low levels yield a high-contrast image, with mainly red/blue colouring. Increasing eDR increases the number of intermediate colours displayed.
Frame rejection (FRe)	0–7	1	5	This function signifies at which signal-to-noise ratio, the whole frame is rejected. Higher levels of FRe signify more rejected frames.
Noise rejection (NRe)	0–7	1	3	This function signifies at which signal-to-noise ratio, an area within the frame is rejected. Higher levels of NRe signify more rejected frames.
Frame Rate (FR)	Min, low, med, high, max	High	Low	FR signifies how often data is collected for cross correlation between frames.
Persistence (Pe)	0–7	7	3	With increasing Pe the temporal resolution decreases, yielding a more constant elastogram with less changes from frame to frame.
Smoothing (Sm)	1–4	3	2	Sm averages pixel colours within the frame creating. By increasing Sm each pixel colour is more dependent on the neighbouring pixel colour.

Only the parameters that differ between the two settings are included in the table.*Derived from Havre et al. [15] # Breast presetting, predefined in the Hitachi RTE-software.

### Evaluation of the elastograms

**Figure 2:**
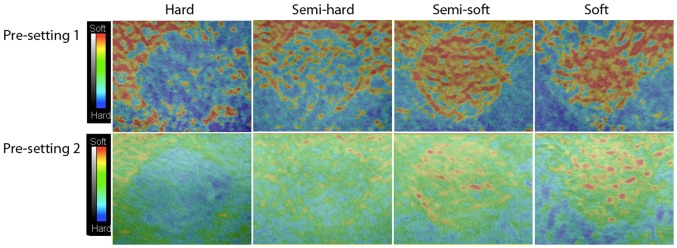
Elastograms of Different Phantom Targets. Elastograms of four different levels of target stiffness with pre-setting 1 and pre-setting 2 in the phantom. The targets are 16.7 mm in diameter. These elastograms were used as teaching examples for the two observers prior to the visual scoring.

**Figure 3:**
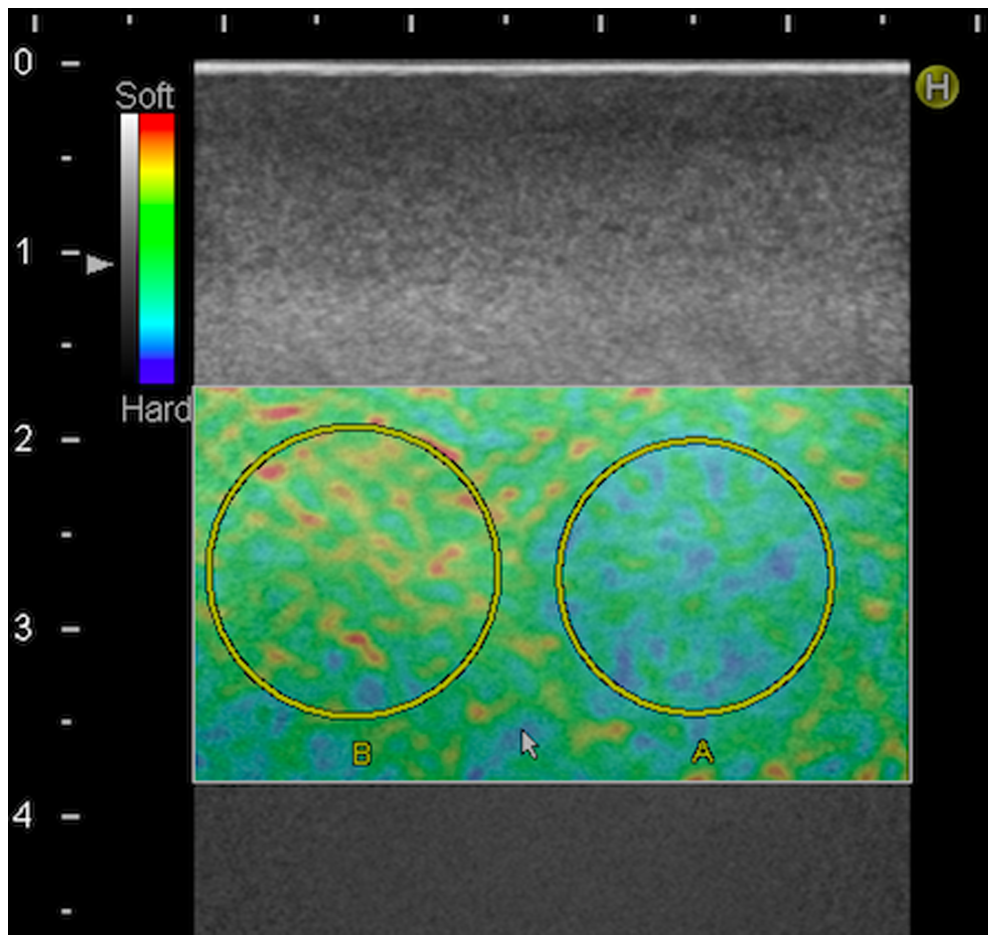
Example of Strain-Ratio Measurement. Placing of ROI A and B for calculation of strain ratios. The target displayed (ROI A) is a 16.7 mm diameter semi hard target assessed using pre-setting 2. Reference ROI (ROI B) is placed in the surrounding medium.

**Figure 4:**
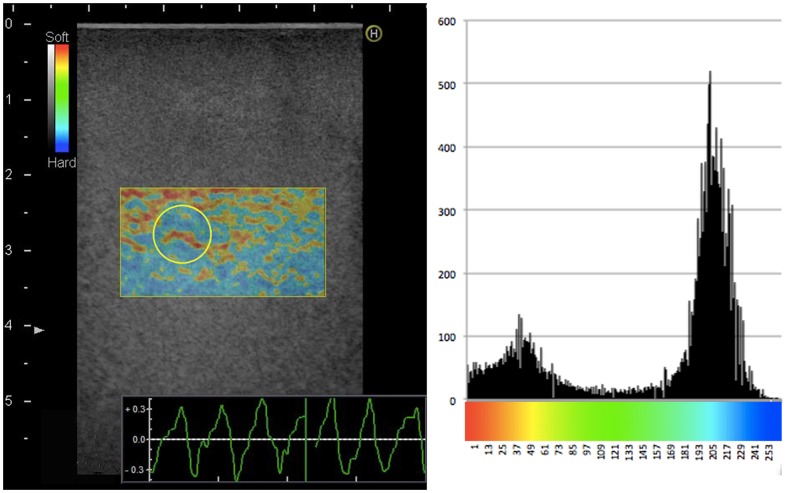
Example of Strain-Histogram Analysis. Strain histogram of a 10.4 mm diameter semi hard target assessed using pre-setting 1, showing (a) the placing of the ROI on the elastogram and (b) the average strain histogram of all frames in the video recorded for the selected target. The x-axis shows the color scale of the elastogram, the y-axis shows the average number of pixels of each color in the elastogram-video.

Evaluation of the elastograms was done by one qualitative and two semi-quantitative methods: visual scoring, strain-ratio measurements and strain-histogram analysis. The visual scoring was done by eight observers independently. Four of the observers (JFC, CE, AS, MBN) had earlier experience with SE, while the remaining four (LL, MT, RRW, CAL), had no prior experience with elastography. The phantom used consisted of cylinders of four different elasticities, therefor a four point scale was used for the analysis. Prior to the evaluation, the observers were shown images of the four levels of target stiffness for each of the two pre-settings analyzed (Figure 2). The evaluations were done blinded and the videos were shown in random order. Still frames were recorded for strain-ratio measurements. The region of interest (ROI) covered the entire lesion and the reference ROI was equally sized and placed in the same depth, according to previous work by Havre et al. [28] (Figure 3). Strain-ratios were calculated as average strain of the surrounding medium divided by the average strain of the target. For each lesion three strain-ratio measurements were performed concurrently with the scanning, as it could not be done off-line, and the mean was calculated. Strain-histograms were performed off-line on uncompressed video files using the free software ImageJ (downloaded at nih.gov) with a plug-in for hue-histogram analysis [29]. For strain histogram analysis ROIs were placed covering the entire target, blinded to the stiffness of the object (Figure 4). ROIs were placed guided by the elastogram and/or the B-mode image. After a ROI was placed, the hue-histogram analysis was performed on the entire video clip. Mean pixel color values were calculated for each frame and afterwards averaged for the entire video clip. One observer performed all calculations. Strain-ratio and strain-histogram analysis yielded continuous numerical data. To transform this into categorical data, optimal cut-offs between the different target stiffness were chosen after data acquisition. For the strain-histograms the cut-offs were changed stepwise with intervals of five units until the highest number of lesions in each group was correctly assessed. For the strain-ratios the intervals were 0.05 units. In a sub-analysis target stiffness was classified as either hard or soft (80 kPa/45 kPa vs. 14 kPa/8 kPa). This was done for comparability with a clinical setting where malignant lesions should be discerned from benign lesions.

### Statistics

The statistical software SPSS version 20 (SPSS, IBM, Chicago, USA) was used for statistical analysis. For the data pooled in two categories, sensitivities and specificities for each method were calculated.

The difference between the methods regarding the number of correctly assessed lesions was calculated using a Chi square test. Linear regression was performed to analyze the impact of the setting, the operator performing the exmination, the size, and the stiffness for each of the three different methods. Backwards elimination of parameters was used until only significant parameters were left in the model. For the visual scorings experience of the observers was also included as an independent variable in the model. Observers were coded as either experienced or inexperienced. The significance level was set at 0.05.

## Results

**Figure 5:**
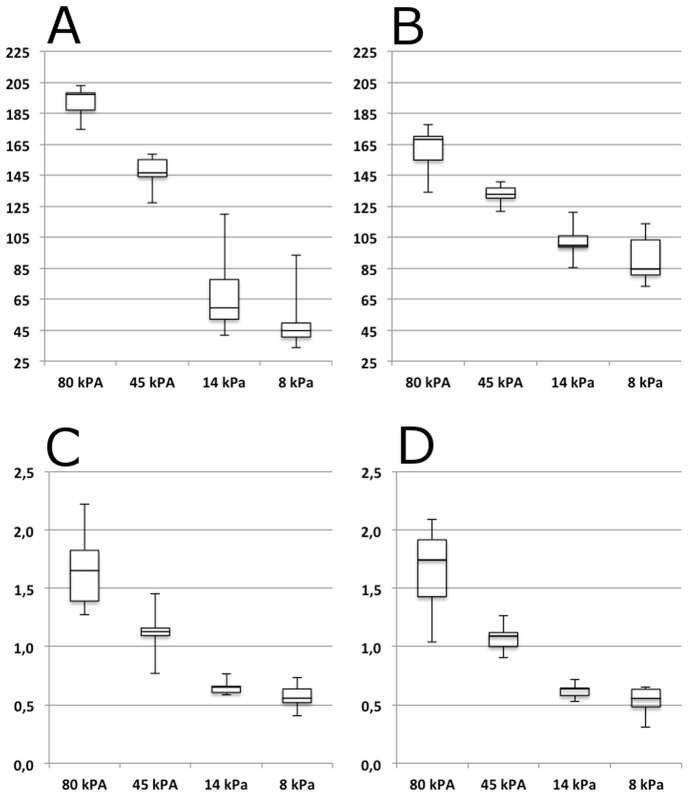
Boxplots of Mean Pixel Values and Mean Strain-Ratios. Box plots of the mean pixel values, unit less (y-axis) of strain-histograms with pre-setting 1 (A) and 2 (B) and mean strain ratios, unit less (y-axis) for pre-setting 1 (C) and 2 (D) for different levels of target stiffness (x-axis). Lower and upper box levels are lower and upper quartiles respectively. The horizontal line within the box marks the median. Whiskers indicate the range.

The optimal cut-offs between the four levels of target stiffness for both strain-ratios and strain-histograms are presented in Table 2. Box plots of the mean pixel values for strain-histograms and mean strain-ratios are displayed in Figure 5.

**Table 2:** A table of the cut-off values for the four levels of target stiffness.

Cut-offs between classes	Strain-histograms * (Presetting 1)	Strain-histograms * (Presetting 2)	Strain-ratios ^#^ (Presetting 1)	Strain-ratios ^#^ (Presetting 2)
1 and 2	**55**	**95**	**0.55**	**0.55**
2 and 3	**125**	**120**	**0.75**	**0.85**
3 and 4	**165**	**145**	**1.20**	**1.30**

* Strain histograms have mean pixel values ranging from 0 to 255. # Strain ratios range from 0 to ∞.

**Figure 6:**
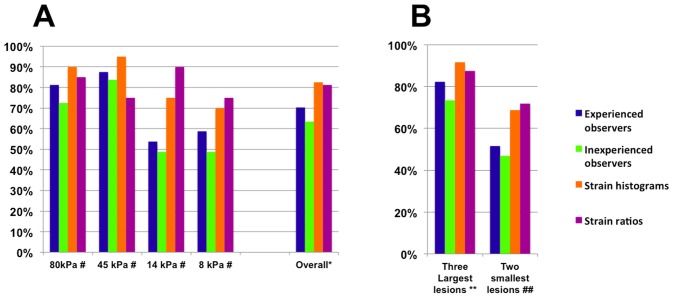
Percentages of Correctly Assessed Targets for Each Method Used. **6a.** Percentages of correctly assessed targets of all diameters. For strain ratios and strain histograms 80 evaluations were performed. For visual scorings 640 observations were performed, 320 by experienced observers, 320 by inexperienced observers respectively. # The bars show mean percentage of correctly assessed targets for each level of target stiffness, with each method of evaluation. * Bars show the mean percentage of correctly assessed targets for all levels of target stiffness for each method of evaluation used. **6b.** Percentages of correctly assessed small and large targets for each method. For strain ratios and strain histograms 80 evaluations were performed. For visual scorings 640 observations were performed, 320 by experienced observers, 320 by inexperienced observers respectively. ** Bars show the mean percentage of correctly assessed targets for the three largest diameters diameters (6.5, 10.4 and 16.7 mm). ## Bars show the mean percentage of correctly assessed targets for the two smallest diameters (2.5 and 4.1 mm).

The percentage of correctly assessed targets with visual scoring, strain-ratios, and strain-histograms is displayed in Figure 6. Figure 6b shows the number of correctly assessed targets when target diameter is larger or smaller than 5 mm respectively. When comparing evaluation of target stiffness with four classes visual score varied significantly from both strain-ratios (p = 0.044 for the experienced observers p = 0.002 for the inexperienced observers) and strain-histograms (p = 0.025 for the experienced observers, p<0.001 for the inexperienced observers) using a Chi-square test. No significant difference was shown when comparing strain-ratios and strain-histograms (p = 0.83) or when comparing the experienced and the inexperienced observers (p = 0.54).

Table 3 reports the sensitivities and specificities of the three methods when using a binary scale (80 kPa/45 kPa vs. 14 kPa/8 kPa, equaling hard and soft). When data was pooled in these two categories, the difference in number of correctly assessed targets between experienced observers and strain ratios and experienced observers and strain histograms were insignificant (p = 0.053 for both) when doing a Chi-square test. The difference between visual scorings by inexperienced observers and strain ratios, and visual scorings by inexperienced observers and strain histograms was significant (p<0.001 for both). The difference between strain ratios and strain histograms was insignificant (p = 1.000), while there was significant difference between experienced and inexperienced observers (p<0.001) using a Chi-square test.

**Table 3:** Percentages of correctly assessed targets.

	Visual scoring	Visual scoring	Strain-histograms	Strain-ratios	Total number of targets evaluated
	Experienced observers (average values) §	Inexperienced observers (average values) §			
80 and 45 kPa (Sensitivities)	38.0 (92.5%)	33.5 (83.8%)	40 (100%)	40 (100%)	40 (100%)
14 and 8 kPa (Specificities)	36.5 (85.0%)	32.0 (80.0%)	39 (97.5%)	39 (97.5%)	40 (100%)
All four elasticities (Accuracies)	74.5 (93.1%)	65.5 (81.9%)	79 (98.8%)	79 (98.8%)	80 (100%)
Two smallest lesions* (All four elasticities)	30.3 (93.0%)	68.8 (71.9%)	31 (96.9%)	31 (96.9%)	32 (100%)
Three largest lesions^#^ (All four elasticities)	44.8 (93.3%)	43.5 (90.6%)	48 (100.0%)	48 (100.0%)	48 (100%)

Numbers of correctly assessed targets when using two classes, 80 kPa/45 kPa vs. 14 kPa/8 kPa and corresponding sensitivities and specificities for each method.

*Diameters of 2.5 and 4.1 mm. # Diameters of 6.5, 10.4, and 16.7 mm. § There were four observers in each group yielding 320 evaluations in all for both experienced and inexperienced obsverers. Numbers listed are the average of the four observers in each group.

Linear regression showed that the presetting and the operator performing the elastography examination had no significant effect on any of the methods evaluated. For strain-histograms only the actual stiffness of the lesion influenced the evaluation (p<0.001). For strain-ratios both the size and stiffness of the target had an influence (p = 0.017 and p<0.001 respectively), as strain-ratios diminished significantly with increasing target diameter For visual scorings size, experience of the evaluator and stiffness had an influence (p<0.001, p = 0.003 and p<0.001 respectively). The inexperienced observers tended to assess the targets as harder than the experienced, while large targets tended to yield harder scorings than small targets.

## Discussion

Our study is the first to report the ability of SE to predict target stiffness in an elasticity phantom. To our knowledge, no previous studies comparing the diagnostic performance of visual scoring with both strain-ratios and strain-histograms have been performed. We showed that strain ratios and histograms are superior to visual scoring in assessing target strain when using four categories of target stiffness. When assessing strain on a binary scale the difference between experienced observers and the semi quantitative methods was not significant.

In a meta-analysis of diagnostic accuracy of elastography in breast cancer diagnosis, strain-ratio analysis was inferior to visual scoring [30]. Our analysis showed that strain-ratios had a higher sensitivity and specificity than visual scoring in a phantom. *In vivo*, the tissues surrounding focal lesions are often quite heterogeneous. The positioning of the reference ROI, for the strain-ratio calculation, may therefore have a large influence on the assessment of stiffness. In our phantom, the surrounding medium was homogenous. This difference may be the reason why we find a better prediction of target stiffness for strain-ratio measurements than for visual scoring. The color scale in strain-histogram analysis is defined by the average strain in the elastography-box and not by a reference ROI. Strain-histograms may therefore provide a better diagnostic tool in inhomogeneous tissues. A meta-analysis of endoscopic SE of focal pancreatic masses showed higher diagnostic accuracy for semi-quantitative assessments (both strain-ratios and strain-histograms) than for visual scoring [31]. This corresponds well with our findings. In endoscopic US transducers and pre-settings differ however from the ones used in the present study which makes direct comparisons difficult.

As the cylinders in the phantom used in the present study had four different levels of stiffness, we applied a four point visual scale for visual scorings. A similar scale using five points has previously been proposed by Itoh et al. [5] and has been widely applied in clinical practice[8].

When the dichotomous stiffness scale was used, we found a significant difference in the sensitivity and specificity of experienced and inexperienced observers doing visual scorings. When using a four point scale, there was no significant difference. This finding may explain some of the variation between different observers reported in previous studies [9], [11], [14], [15]. To our knowledge no studies have investigated the effect of experience on visual scoring in SE previously.

Our phantom study has illustrated the impact of several parameters on three different methods of evaluation of strain elastography. These parameters must be taken into account, when performing elastography in a clinical setting.

In a study by Havre et al. the influence of different scan parameters on the elastogram quality was evaluated when using a visual scale on a phantom [15]. They concluded that a pre-setting corresponding to our pre-setting no. 1 was optimal in a phantom. We found no significant influence from the pre-settings on the prediction of target stiffness. Thus, in a phantom, the perceived visual quality may change to a certain degree without altering the prediction of target strain. Pre-setting no. 1, with a low elasticity dynamic range, correlates well with the phantom used because tissues are depicted as either hard (blue) or soft (red). Pre-setting no. 2, with higher elasticity dynamic range, may better depict the variance of stiffness within in-vivo tissue. No studies on the influence of elastography pre-setting on diagnostic performance have been published previously.

We were unable to visualize any of the four cylinders at their smallest diameters (1.6 mm). No studies have been conducted on SE resolution, though some studies have shown good diagnostic performance of SE in small breast tumors [32], [33]. Studies of enlarged lymph nodes in EUS elastography have shown that a trained observer has equal diagnostic performance as strain-histogram and strain-ratios, when lesions are large [22]. In agreement with this, we found a trend towards improvement of visual scoring in large targets compared with small targets. We also found that size influenced the assessment of target stiffness in visual scoring and in strain ratio assessments significantly.

The smaller phantom targets were difficult to localize on B-mode. This may have caused a lower prediction of target stiffness in the phantom than would have been the case *in vivo*, where lesions are normally first seen on B-mode. Strain-ratio measurements could not be done blinded to target strain, they may therefore show better diagnostic performance than if done blinded.

No studies comparing strain-based elastography with SWE or ARFI have been published. ARFI-measurements in a phantom have been studied [34], [35], but none has reported on diagnostic performance of either SWE or ARFI in a phantom.

In conclusion strain-histograms and strain-ratios are superior to visual scoring in assessing target stiffness in a phantom. Target size had a significant influence on strain-ratio assessments and visual scorings. There was no effect of target size on strain-histograms. A significant difference when comparing experienced and inexperienced observers was shown when assessing target stiffness using a binary scale. The pre-setting used had no significant influence on the scorings.
